# Safeguarding the physical health of people with severe mental disorders during the COVID-19 pandemic

**DOI:** 10.1192/bjb.2020.92

**Published:** 2020-10

**Authors:** Shuo Zhang, Jayati Das-Munshi, Graham Thornicroft

**Affiliations:** CT2, South London and Maudsley NHS Foundation Trust, UK; email: Shuo.zhang@kcl.ac.uk; Consultant Psychiatrist and Clinician Scientist Fellow, Department of Psychological Medicine, IOPPN, King's College London, UK; Professor of Community Psychiatry, Centre for Global Mental Health and Centre for Implementation Science, IOPPN, King's College London, UK

The coronavirus disease 2019 (COVID-19) pandemic is putting unprecedented stress on global healthcare systems. Psychiatrists have also seen great changes to their day-to-day practice with a move towards telephone and video consultations alongside general practice and secondary care colleagues. As we move towards operating in these new ways for the foreseeable future, it is likely that COVID-19 will further exacerbate multilevel risk factors for excess mortality in people with severe mental disorders (usually understood to include people with psychosis, bipolar disorder or major depressive disorder).

People with SMD already have a 2–3 times higher premature mortality rate, accounting for a 10–20-year reduction in life expectancy, mediated through increased exposure to risk factors for non-communicable diseases, such as smoking, harmful use of alcohol, sedentary behaviour, iatrogenic effects of medications and inequitable access to healthcare services.^1^ Those with SMD also often receive poor quality care, including health promotion and prevention, screening and treatment.

Individuals at higher risk for severe COVID-19 infection and mortality are people aged over 60; with underlying conditions such as obesity, hypertension, diabetes, cardiovascular disease, or chronic respiratory disease; and those who smoke.^2^ For other infectious diseases, people with SMD are likely to be at increased risk of: (a) exposure to the disease; (b) accessing less effective healthcare; and (c) increased vulnerability for significant morbidity and mortality.^1^

Although there are overlaps with pre-existing multilevel risk factors,^3^ there are some important differences. For individuals with SMD, disorder-specific factors of COVID-19 such as early symptoms being common and non-specific could delay diagnosis, and it is possible that people with SMD may be less able to self-monitor and raise concerns if their condition deteriorates. Furthermore, COVID-19 has the potential to mimic signs and symptoms seen in severe clozapine-associated complications, such as neutropenic sepsis and myocarditis, which can be difficult to clinically differentiate from severe COVID-19.^2^ We anticipate that health-related behaviours, such as tobacco use and associated higher prevalence of underlying lung disease in the SMD population, will increase the risk of COVID-19 complications and deaths from pneumonia.

Individual vulnerabilities are exacerbated by health system factors such as absence of relevant shared guidelines for the management of COVID-19 from physical health and mental health bodies, diversion of resources from mental health settings, high rates of COVID-19 illness within the health workforce, and the challenges of infection control management in mental healthcare settings, exacerbated by global shortages of personal protective equipment (PPE).

People with SMD have continued higher exposure to sociocultural risk factors including experiences of stigma and discrimination, living in deprived neighbourhoods, and limited family and community resources.^3^ At present the impact of these factors within the context of the COVID-19 pandemic is unclear.

We suggest the following measures to address individual, facility and health system determinants of health.

Individuals should be supported with infection prevention, for example the direct provision of education about handwashing, social distancing and the signs and symptoms of COVID-19 along with health promotion strategies such as smoking cessation or harm reduction, reducing drug and alcohol use, and optimising conditions such as diabetes mellitus, chronic respiratory conditions and cardiovascular health. People with SMD should maintain contact with mental healthcare teams and receive ongoing review of mental health needs. At present although there has been some specific guidance on supporting people with cognitive impairment and dementia,^4^ it remains unclear what the impact of the pandemic may be on people with SMD, a group who may be especially vulnerable because of pre-existing social isolation, which may be further exacerbated by social distancing measures.

Staff at mental health community and residential facilities should have equal access to PPE and training on infection control in order to reduce the risks of infection. Urgent reviews of visitor policies, and assurance of sick pay for self-isolating staff are also needed.

Further consideration is needed to optimise effective delivery of care when mental and physical health staff reduce routine face-to-face meetings, and to minimise disruptions in the supply of medication and routine monitoring of medications such as clozapine and lithium. People with SMD may need additional support in accessing services when community health centre staff adopt new ways of working, such as telephone or video consultations.

People with SMD should be offered the same level of treatment for physical healthcare in line with the principles of non-discrimination, the Right to Health, and the fundamental demand of the United Nations Sustainable Development Goals,^5^ namely to ‘leave no-one behind’.
